# Work Engagement and Well-being Study (SWELL): a randomised controlled feasibility trial evaluating the effects of mindfulness versus light physical exercise at work

**DOI:** 10.1136/bmjment-2023-300885

**Published:** 2024-02-28

**Authors:** Maris Vainre, Tim Dalgleish, Peter Watson, Christina Haag, Quentin Dercon, Julieta Galante, Caitlin Hitchcock

**Affiliations:** 1 Medical Research Council Cognition and Brain Sciences Unit, Cambridge University, Cambridge, UK; 2 Institute of Psychology, University of Tartu, Tartu, Estonia; 3 Institute for Implementation Science in Health Care, University of Zurich, Zurich, Switzerland; 4 Epidemiology, Biostatistics and Prevention Institute, University of Zurich, Zurich, Switzerland; 5 Institute of Mental Health, University College London, London, UK; 6 Department of Psychiatry, University of Cambridge, Cambridge, UK; 7 Contemplative Studies Centre, University of Melbourne, Melbourne, Victoria, Australia; 8 Melbourne School of Psychological Sciences, University of Melbourne, Melbourne, Victoria, Australia

**Keywords:** Adult psychiatry

## Abstract

**Background:**

Mindfulness-based programmes (MBPs) are increasingly offered at work, often in online self-guided format. However, the evidence on MBPs’ effect on work performance (WP) is inconsistent.

**Objective:**

This pragmatic randomised controlled feasibility trial assessed procedural uncertainties, intervention acceptability and preliminary effect sizes of an MBP on WP, relative to an alternative intervention.

**Methods:**

241 employees from eight employers were randomised (1:1) to complete a 4-week, self-guided, online MBP or a light physical exercise programme (LE)(active control). Feasibility and acceptability measures were of primary interest. WP at postintervention (PostInt) was the primary outcome for preliminary assessment of effect sizes. Secondary outcomes assessed mental health (MH) and cognitive processes hypothesised to be targeted by the MBP. Outcomes were collected at baseline, PostInt and 12-week follow-up (12wFUP). Prospective trial protocol: NCT04631302.

**Findings:**

87% of randomised participants started the course. Courses had high acceptability. Retention rates were typical for online trials (64% PostInt; 30% 12wFUP). MBP, compared with the LE control, offered negligible benefits for WP (PostInt (*d*=0.06, 95% CI −0.19 to 0.32); 12wFUP (*d*=0.02, 95% CI −0.30 to 0.26)). Both interventions improved MH outcomes (*d*s=−0.40 to 0.58, 95% CI −0.32 to 0.18); between-group differences were small (*d*s=−0.09 to 0.04, 95% CI −0.15 to 0.17).

**Conclusion:**

The trial is feasible; interventions are acceptable. Results provide little support for a later phase trial comparing an MBP to a light exercise control. To inform future trials, we summarise procedural challenges.

**Clinical implications:**

Results suggest MBPs are unlikely to improve WP relative to light physical exercise. Although the MBP improved MH, other active interventions may be just as efficacious.

**Trial registration number:**

NCT04631302.

**WHAT IS ALREADY KNOWN ON THIS TOPIC**

- Mindfulness-based programmes (MBPs) have been shown to improve mental health when compared with passive control groups, and there is some indication that they may also improve work performance.

**WHAT THIS STUDY ADDS**

- This trial is the first to compare an MBP to an active comparison intervention on their effects of work performance. This early-phase trial determined the feasibility of a later stage efficacy trial.

**HOW THIS STUDY MIGHT AFFECT RESEARCH, PRACTICE OR POLICY**

- We found that an online MBP is likely to yield no benefit compared with a light physical exercise online programme, either for work performance or mental health. Public health recommendations on offering MBPs should consider comparative effectiveness of alternative approaches, along with users’ preferences.

## Background

Public health guidance in several countries encourages employers to support the physical activity (eg, ref 1 2) and mental health (eg, ref 2 3) of staff. Employers, too, are increasingly seeking to support employees’ health and well-being by incorporating mindfulness-based programmes (MBPs) into their well-being package,^4–7^ as recommended in official guidance.^3^ MBPs aim to improve attention and self-regulation through training the ability to maintain awareness of the present moment.^8^ MBPs also cultivate compassion^9 10^ by fostering a detached self-perspective and thus training recipients to decentre from psychological stressors. There is existing evidence that MBPs have several mental health benefits, when compared with passive face-to-face control groups (usually waitlist control), including reduction in symptoms of anxiety, depression and stress in community populations.^11^

In the workplace, mindfulness practices enhance mental health and work performance, making them appealing to employers compared with other well-being strategies such as other well-being interventions or organisational changes.^12^ MBPs, when available online, align with remote work trends and offer logistical advantages like scalability, cost-effectiveness and flexible access without scheduling conflicts, benefiting organisational well-being initiatives.

Yet, the empirical data on whether MBPs improve work performance remain equivocal. A recent systematic review suggests that work performance is rarely assessed in trials investigating the outcomes of MBPs at work.^13^ When work performance is assessed, a wide range of operationalisations are used, ranging from resilience^14 15^ and work engagement^16–19^ to absenteeism/presenteeism.^19–24^ It is thus unclear whether MBPs: (a) improve an individual’s perceived ability to complete their job; and (b) are more effective than other work-based interventions. Perceived ability to engage in work is a key influence on individual-level experience (eg, in line with the WHO definition of health^25^) and on the economy (eg, through decisions not to engage in the workforce due to low self-efficacy). Clarifying these points will guide informed MBP adoption decisions at the organisational level.

MBPs could improve work performance through two pathways. First, MBPs have a demonstrated ability to reduce symptoms of poor mental health which could enhance work performance. Better mental health is likely to impact several aspects of work performance,^26^ such as improving resilience and work engagement, and reducing absenteeism/presenteeism. A second potential pathway is via cognitive control, that is, through the ability to self-regulate at work to allow prioritisation of current goals.^27 28^ According to recent meta-analyses,^29–32^ mindfulness training, compared with passive control groups, could enhance cognitive control (Hedge’s *g*=−0.03 to 0.42), but it has yet to be determined whether improved cognitive skills transfer to work performance. Furthermore, existing research has focused on the impact of mindfulness on cognitive control over affectively benign information. Yet, much of the everyday mental activity that we seek to regulate while at work is emotionally positive or negative.^33 34^ Reduced ability to inhibit internal affective stimuli (eg, remembering an argument with your spouse) may interfere with the ability to maintain focus on tasks at work (eg, writing a paper). As mindful meditation is proposed to train the ability to move away from thoughts and images, in this study we sought to explore whether practising mindfulness may particularly enhance cognitive control over affective mental events.^9^

If cognitive control is a key pathway through which MBPs work, then the most likely domain of work performance^26^ to improve is task performance, or the quantity and quality of work. This domain has been less frequently assessed, compared with other types of work performance.^35^ It is difficult to assess task performance in a way that would allow for a comparison between different job roles and industries.^36^ However, beliefs in one’s ability to complete job-related tasks have been shown to predict improved work performance,^37^ particularly task performance,^38^ with some suggestions that this effect may occur via cognitive control.^39^

In sum, it is currently unclear whether MBPs (particularly online, self-guided MBPs) can improve work performance, the mechanisms through which any such effect may occur, and whether their effects are superior to those of other workplace interventions. A better understanding of the effects of MBPs on work performance could lead to immediate applications in workplaces. Further investigation of potential mechanisms of action could also improve our attempts to assess MBPs by designing and selecting more stringent outcome measures and control interventions, and guiding decisions regarding for whom MBPs may be most effective, and in which context.^40^

A definitive randomised controlled trial (RCT) is thus needed to evaluate the effect of an MBP on work performance and whether cognitive control is its mechanism. This is best done via an RCT with an active control group that would control for other potential pathways of effect (ie, mental health). However, little is known about the comparative effects of an MBP against a control condition that also improves mental health.^35^ Prior reviews indicate that the current quality of the evidence is low, and Consolidated Standards of Reporting Trials (CONSORT)-standard, pre-registered trials are needed.^11 13 35^ Here, we aim to determine feasibility of a phase II RCT comparing an MBP to light physical exercise. This feasibility trial aimed to clarify procedural uncertainties. Given effect sizes are difficult to predict, we complete a preliminary investigation of the relationships between an MBP, work performance and the proposed mechanisms of action, to inform a later stage trial.

### Objective

This feasibility trial aimed to clarify methodological uncertainties and determine feasibility of a later stage randomised controlled efficacy trial to evaluate the effects, and underpinning mechanisms of action, of online self-guided MBPs on work performance.^41–43^ Participants were randomised to complete either an online, self-guided MBP, or a light physical exercise active control intervention designed to control for mental health benefits on work performance as well as other non-specific effects such as participating in a structured intervention requiring engagement with the body. There is no evidence that low-intensity exercise improves cognitive control in healthy working-age adults.^44^ This feasibility trial:

- Sought to resolve design and procedural uncertainties in advance of a later stage trial.
- Assessed the acceptability of both the MBP and control interventions.
- Estimated the between-group effect size for the effect of the MBP, relative to an active control on our primary outcome of work performance (at postintervention), in order to inform power calculations for a later phase trial.
- Estimated the effect of cognitive control as mediator of the effect of MBP on work performance.

## Methods

This trial adheres to CONSORT guidelines for randomised pilot and feasibility trials^45^ (online supplemental material 1a). The trial was prospectively registered at ClinicalTrials.gov (NCT04631302). Full methodological details can be found in the published protocol.^46^

10.1136/bmjment-2023-300885.supp1 Supplementary data

### Study design and participants

Randomisation was conducted at the participant level on a 1:1 ratio. We recruited participants by first approaching organisations with employees primarily engaged in desk-based occupations. The participants were able to start their course every Monday from 1 March to 28 June 2021 and from 4 to 25 October 2021. The participating employers distributed information about the study through their usual internal media channels (emails, MS Teams, Slack, etc).

Inclusion criteria (all self-reported) were being a current employee of a participating employer, and based in the UK. We did not specifically seek out healthy employees, however, we suggested participants not to participate if they were currently on long-term leave, currently suffering from severe anxiety, depression, hypomania/mania or other severe mental illness, having experienced a recent bereavement or major loss, having already completed a mindfulness course or having meditated more than 10 hours in the past 10 years. All participants provided written informed consent. There were no incentives for completing the intervention. Participants received retail vouchers for completing the postintervention (£10) and 12-week (£15) assessments and were encouraged to complete the assessments regardless of how much (if any) of the intervention they had completed.

### Sample size

As this was a feasibility trial, sample size was not guided by a formal power calculation to estimate effect size. We aimed to recruit 240 participants,^46^ anticipating this would yield 128 participants (64 per arm) at postintervention and 68 (34 per arm) at follow-up, given high attrition rate experienced by trials completed online.^47^ This sample size is standard for feasibility trials in the UK^48^ and provides enough data to evaluate procedural uncertainties and acceptability and to provide a range of effect size estimates on our primary outcome.

### Intervention arm: *Be Mindful* MBP

Participants in the MBP arm completed the 4-week *Be Mindful* pre-recorded and fully automated online course by Wellmind Media.^49^ Materials and instructional videos were accessed through a website (http://www.bemindfulonline.com) (see ref 50, online supplemental material 1b and online supplemental material 2).

10.1136/bmjment-2023-300885.supp4 Supplementary data

### Active control arm: light physical exercise

The 4-week pre-recorded and fully automated online light physical exercise (LE) programme aimed to enhance mobility, alleviate stiffness, stimulate blood flow and prevent pain or repetitive strain injuries that may arise from tasks typical in office settings (see online supplemental material 1c and online supplemental material 2 and ref 51). This control arm matched the mindfulness arm in overall time commitment and frequency of interaction with the participant.^46^ It also encouraged use of short breaks throughout the workday to focus on well-being, replicating the mindfulness programme. A previous study has demonstrated the course to have active benefits for mental health,^51^ thus allowing us to control for non-specific and mental health intervention effects.

### Outcomes

We collected demographics and work-related details at baseline. Assessments were completed at baseline, postintervention (primary endpoint) and 12-week follow-up. Additionally, participants were invited to complete a brief questionnaire each day they worked. Participant-reported outcomes were collected online via REDCap^52^ and jsPsych^53^ hosted on JATOS.^54^

#### Feasibility and acceptability

The acceptability of the interventions was assessed by uptake at recruitment, retention and monitoring adherence to the intervention protocol, indexed via tracking participants’ logins to their respective intervention website. The design and procedural feasibility of a later stage trial was determined by monitoring recruitment of both organisations and participants and trial retention, evaluating the willingness of the participants to be randomised, intervention contamination (ie, participants completing exercises that corresponded to the other arm of the trial or talking about the course with participants in the other arm), course preferences and outcome measures’ completion rates.

#### Primary outcome: work performance

To estimate the likely effect size for a later phase trial, work performance was measured by using the 25-item Work Role Functioning Questionnaire (WRFQ) version 2,^55^ see online supplemental material 2. The questionnaire has not been validated in English, however, validations of Dutch, Spanish and Norwegian versions have shown good internal consistency (Cronbach’s α=0.7–0.9)^55–57^ and test–retest reliability (intraclass correlation coefficient (ICC)=0.66, 95% CI 0.54 to 0.76 for the total score).^55^ In the current study, Cronbach’s α=0.93. Our primary endpoint was postintervention.

#### Secondary outcomes

##### Work-related outcomes

Participants were asked to report whether their ability to work was impacted by physical health problems, mental health problems, other health problems, no problems or prefer not to say. Those who reported any health problems were asked to fill in the Work and Social Adjustment Scale.^58^ To index daily fluctuations that may occur in work engagement, participants were invited to complete the 5-item version of the WRFQ^59^ at 15:00 each working day, to reflect on work performance that day. Items are scored as per the full WRFQ.

##### Cognitive control mechanisms

Two online computerised cognitive control tasks were written in JavaScript. Affective cognitive control was assessed using the affective stop-signal task^60 61^ (see online supplemental material 2). The main outcome of interest was the stop signal reaction time (SSRT; milliseconds), after excluding trials where the reaction time was improbably short (250 ms or less) or long (above 3000 ms) and where the stop signal delay was below 50 ms, as recommended by the task authors.^62^

Participant’s ability to track dynamic changes in their environment and alter their response strategies was measured using an affective modification of the probabilistic reversal learning task^63 64^ (see online supplemental material 2). The main outcome of interest was change in learning performance indexed via the overall proportion of correct responses.

##### Other outcomes of interest

The Perceived Stress Scale measured the extent to which the individual perceives events as uncontrollable and overwhelming.^65^ The Patient Health Questionnaire 9 (PHQ-9)^66^ was used to assess depression symptoms. The Generalized Anxiety Disorder 7-item Scale (GAD-7)^67^ assessed anxiety symptoms. The Experiences Questionnaire^68^ measured decentring. The Mindful Attention Awareness Scale (MAAS)^69^ assessed self-reported dispositional mindfulness. We also planned to use the Short Warwick-Edinburgh Mental Well-Being Scale.^70^ However, due to a technical error we were unable to obtain the data.

### Randomisation and masking

After completion of the baseline assessment, participants were randomly assigned to either the mindfulness or light physical exercise arm, stratified by employer. The randomisation process was automated in REDCap.^52 71^ The study manager (MV) clicked a button that randomised the participant using a prespecified allocation table (created with randomizeR package^72^ in R with randomly selected block sizes^73^). The allocation table could not be edited once data collection had begun, and concealed the allocation process from the researchers. An automated email informed the participant of their allocation and detailed how to access the relevant course. The randomisation code is available at GitHub.^74^ Neither the participants nor the study manager was blind to intervention allocation, although the participants were not told which intervention is considered to be the control and study materials introduced both courses equivalently. The primary analysis was completed by a statistician (PW) blinded to intervention allocation.

### Statistical methods

Data were analysed in *R* 75 using RStudio^76^ (online supplemental material 2). As per our pre-registration, primary and secondary outcomes were analysed using the intention-to-treat principle. We ran multiple linear regression models with the *miceadds* package,^77^ using separate models to compare postintervention and follow-up scores between trial arms, adjusted for baseline and employer. The postintervention questionnaire data analyses including our primary outcome (except MAAS and decentring) were completed by an independent statistician blinded to intervention allocation (PW). The remaining secondary outcomes at postintervention and all follow-up analyses were completed by MV.

## Findings

The raw data along with the code to analyse it is available online.^78^

### Feasibility and acceptability

#### Recruitment feasibility

Eight employers and 241 employees participated in the trial. The median number of staff members per participating employer was 2130 (range: 180–7500, total of 20 966 UK-based employees). The percentages of those staff members who agreed to take part and were randomised were modest (median: 0.91%, range: 0.27–2.85%). Compared with other industries, a larger proportion of local authority employees joined the study (M=2%; SD=0.74 vs M=0.58%; SD=0.34). For sample characteristics at baseline, see table 1.

**Table 1:** Baseline characteristics

Characteristic	Mindfulness (n=122)	Light exercise (n=119)
Gender		
Female (%)	105 (86.1)	99 (83.2)
Age, M (SD)	44.22 (11.13)	45.04 (10.21)
Employer (%)		
Engineering	4 (3.3)	4 (3.4)
Higher education	1 (0.8)	1 (0.8)
Local authority 1	7 (5.7)	6 (5)
Local authority 2	69 (56.6)	69 (58)
Local authority 3	24 (19.7)	23 (19.3)
Publishing	4 (3.3)	2 (1.7)
Secondary education	1 (0.8)	1 (0.8)
Technology	12 (9.8)	13 (10.9)
Ethnicity (%)		
Asian	6 (4.9)	9 (7.6)
Mixed or multiple	5 (4.1)	2 (1.7)
Other	2 (1.6)	3 (2.5)
Prefer not to answer	1 (0.8)	–
White	108 (88.5)	105 (88.2)
Education		
Degree (%)	87 (71.3)	74 (62.2)
Caring responsibilities		
Yes (%)	43 (35.2)	46 (38.7)
Condition that affects the ability to focus		
Yes (%)	9 (7.4)	10 (8.4)
Any experience with meditation		
Yes (%)	47 (38.52)	53 (44.54)

#### Intervention acceptability

**Figure 1:**
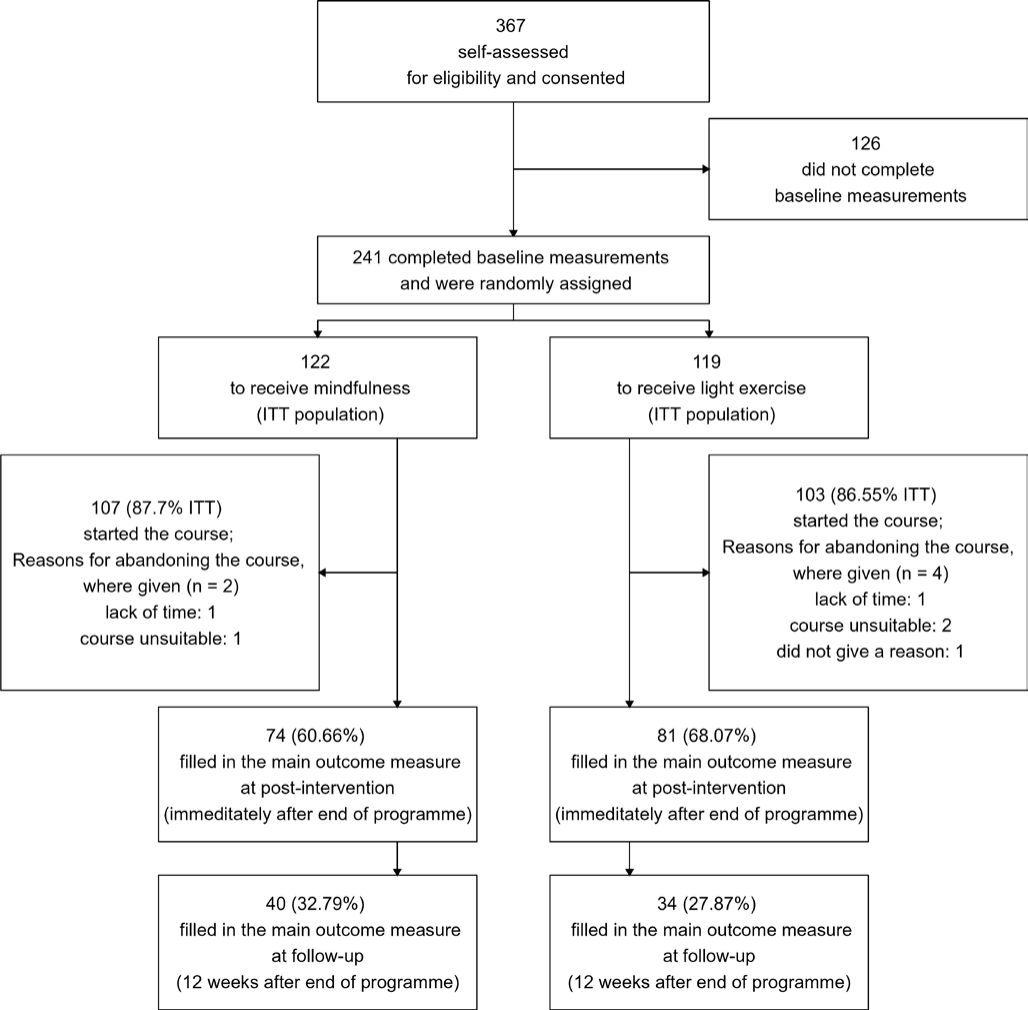
Consolidated Standards of Reporting Trials (CONSORT) diagram illustrating participant flow. ITT, intention to treat.

Eighty-seven per cent of randomised participants started the course (87.7% in mindfulness, 86.55% in light physical control). The retention rates for outcome measure collection were 64% (60.66% in mindfulness, 68.07% in light physical exercise) at postintervention and 30% (32.79% in mindfulness, 27.87% in light physical exercise) at follow-up. Six participants decided to abandon the programme but agreed to provide outcome measures at time points prespecified in the protocol.^46^ Of those six, three participants found the assigned programme unsuitable (one in mindfulness, two in light exercise). No participant requested to withdraw from the study (for CONSORT diagram see figure 1). Across both interventions, the median length of intervention engagement was 3 weeks out of four (IQR=2). At postintervention, participants in the light exercise programme showed a greater desire to have been assigned to mindfulness, while the participants in the mindfulness arm did not show a strong preference either way (W=3523, p=0.02). For further details, see online supplemental material 3: table 1.

10.1136/bmjment-2023-300885.supp5 Supplementary data

#### Contamination

At postintervention, mindfulness participants reported to have talked about their course with light exercise participants slightly more frequently (M=5.07; SD=14.63 on a 0…100 scale) than the other way around (M=2.81; SD=5.85), the difference was not statistically significant (W=2911, p=0.93).

Participants in both intervention arms reported similar levels of weekly exercise at both postintervention (p=0.61) and at follow-up (p=0.94) (for details, see online supplemental material 3: table 1). At postintervention, while participants in both arms had practised mindfulness up to 3 hours/week, those assigned to mindfulness were more likely to have done so: 54.91% (mindfulness) versus 16.8% (light exercise) (χ^2^(2, 246)=12.8, p=0.002).

### Effect size estimation for primary outcome: work performance

**Figure 2:**
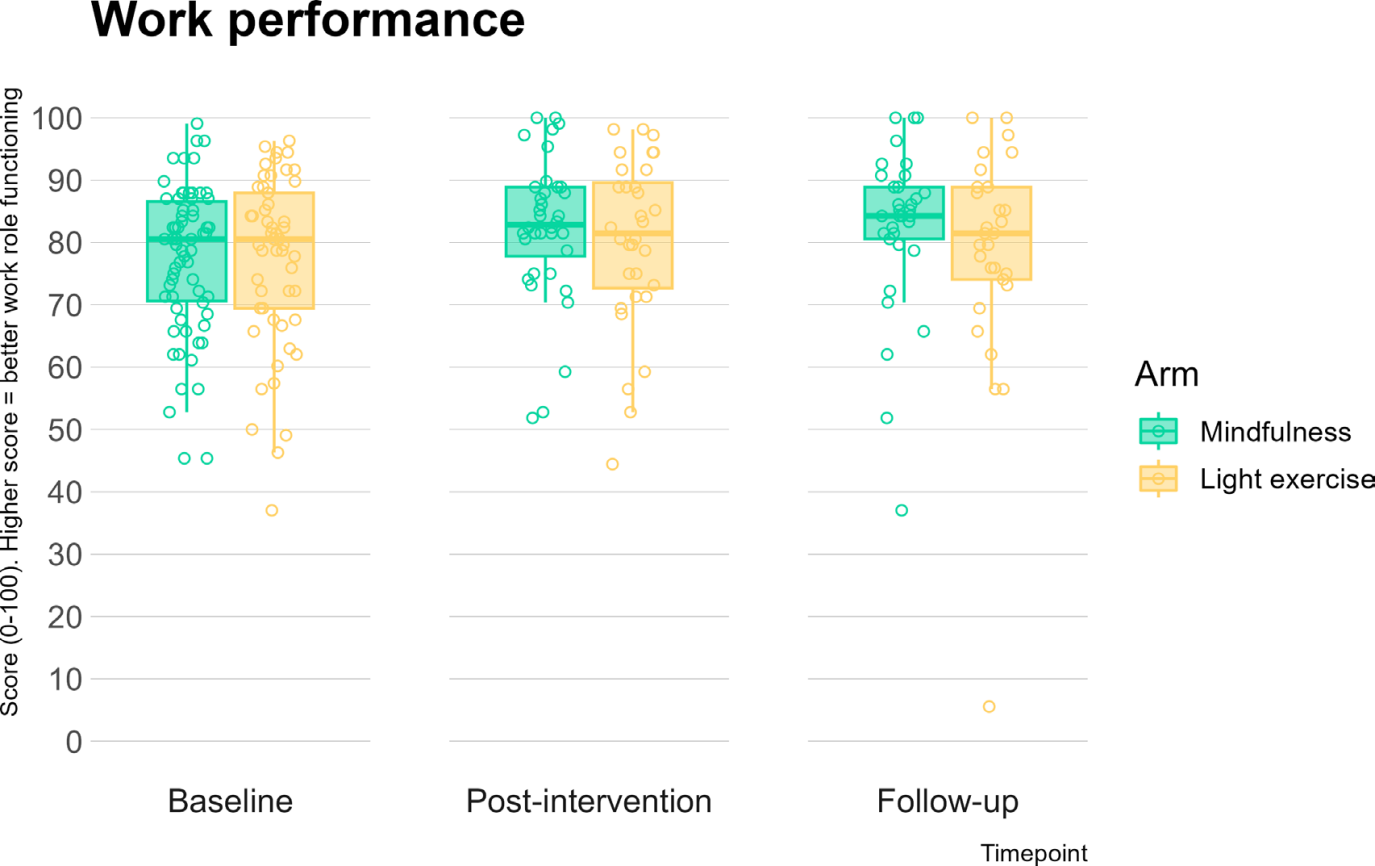
Work Role Functioning Questionnaire total score at baseline, postintervention and 12-week follow-up compared across the two study arms.

The intention-to-treat analysis indicated that, adjusting for baseline and employer, there was a negligible effect size for the difference between the mindfulness and light exercise arms in work performance at our primary endpoint of postintervention (*d*=0.06, see figure 2; online supplemental material 3: table 2). As expected for a feasibility trial, this difference was not statistically significant (t(237)=0.49, p=0.63).

### Effect size estimation for secondary outcomes

#### Further work-related outcomes

When examining preintervention to postintervention change, trivial, non-significant effect sizes were observed for all participants, indicating minimal improvement in work performance, regardless of intervention allocation (*d*=0.10, p=0.28). Similar effect sizes were observed for change from preintervention to follow-up (*d*=0.14, p=0.12). The light exercise participants reported more overtime hours than those in the mindfulness programme (*d*=0.22, p=0.09) at postintervention, along with more frequent health problems (*d*=0.20, p=0.11), with small effect sizes. At follow-up, the difference between mindfulness and light exercise on WRFQ total score was trivial (*d*=0.02, p=0.91, see figure 2; online supplemental material 3: table 2).

We found daily monitoring a fraught approach to index work performance. On average, participants completed the daily questionnaire on fewer than half of the 28 days (mindfulness: M=12.2, SD=6.95, min=1, max=27; light exercise: M=9.95, SD=7.55, min=1, max=26). Some participants did not respond to any of the daily monitoring questionnaires (mindfulness n=24 (19.67%), light exercise n=16 (13.45%)). Across the 28 days, the average work functioning score improved across arms (the effect of day: beta=0.20, SE=0.05, t(25.96)=4.03, p=0.0004), with a negligible effect size for the between-arm difference (beta=0.15, SE=0.94, t(163.52)=0.16, p=0.873, online supplemental material 3: figure 4).

#### Mental health

**Figure 3:**
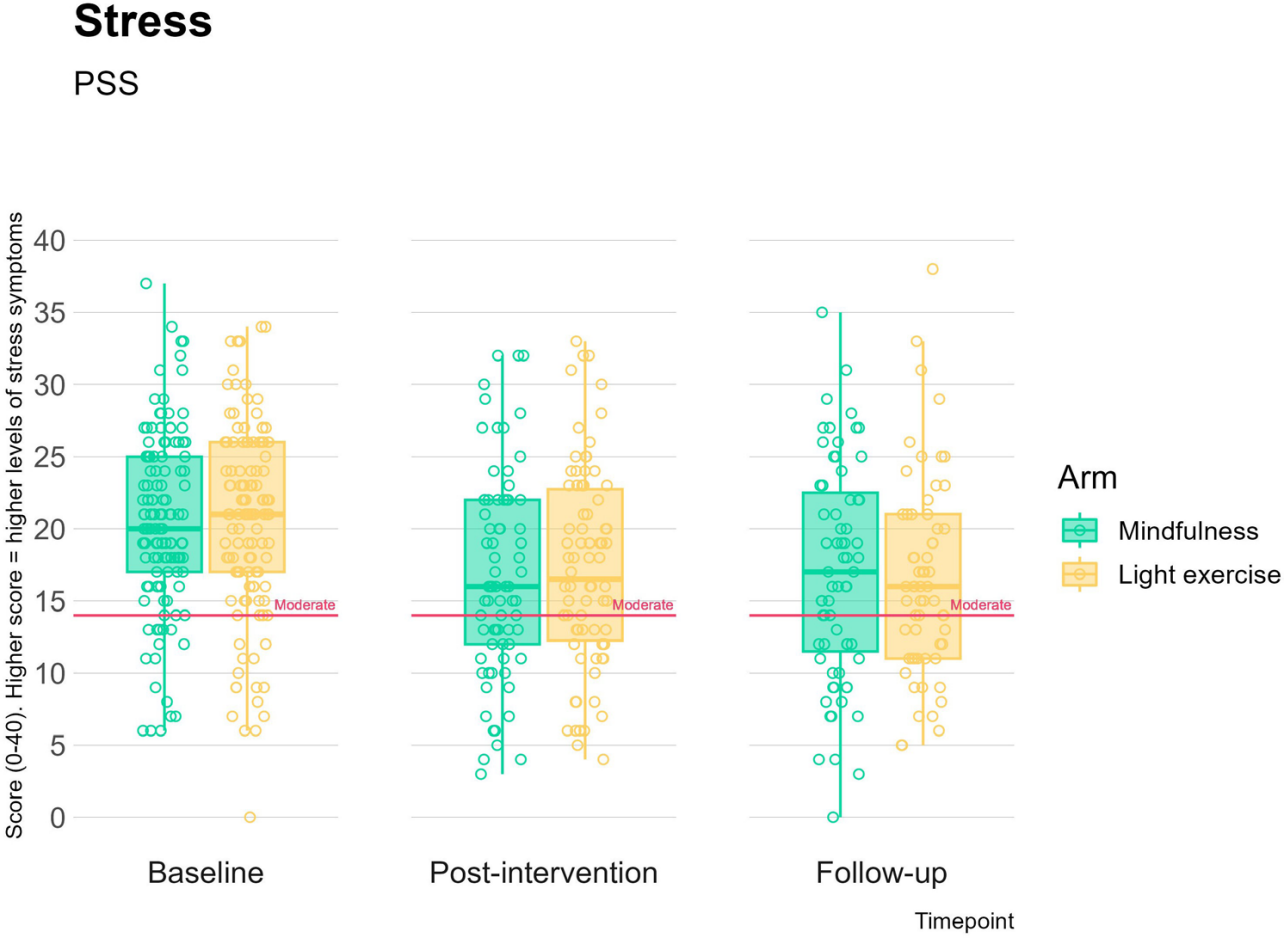
Perceived Stress Scale (PSS) score at baseline, postintervention and 12-week follow-up compared across the two study arms. The pink lines indicate the cut-off score for moderate stress.

**Figure 4:**
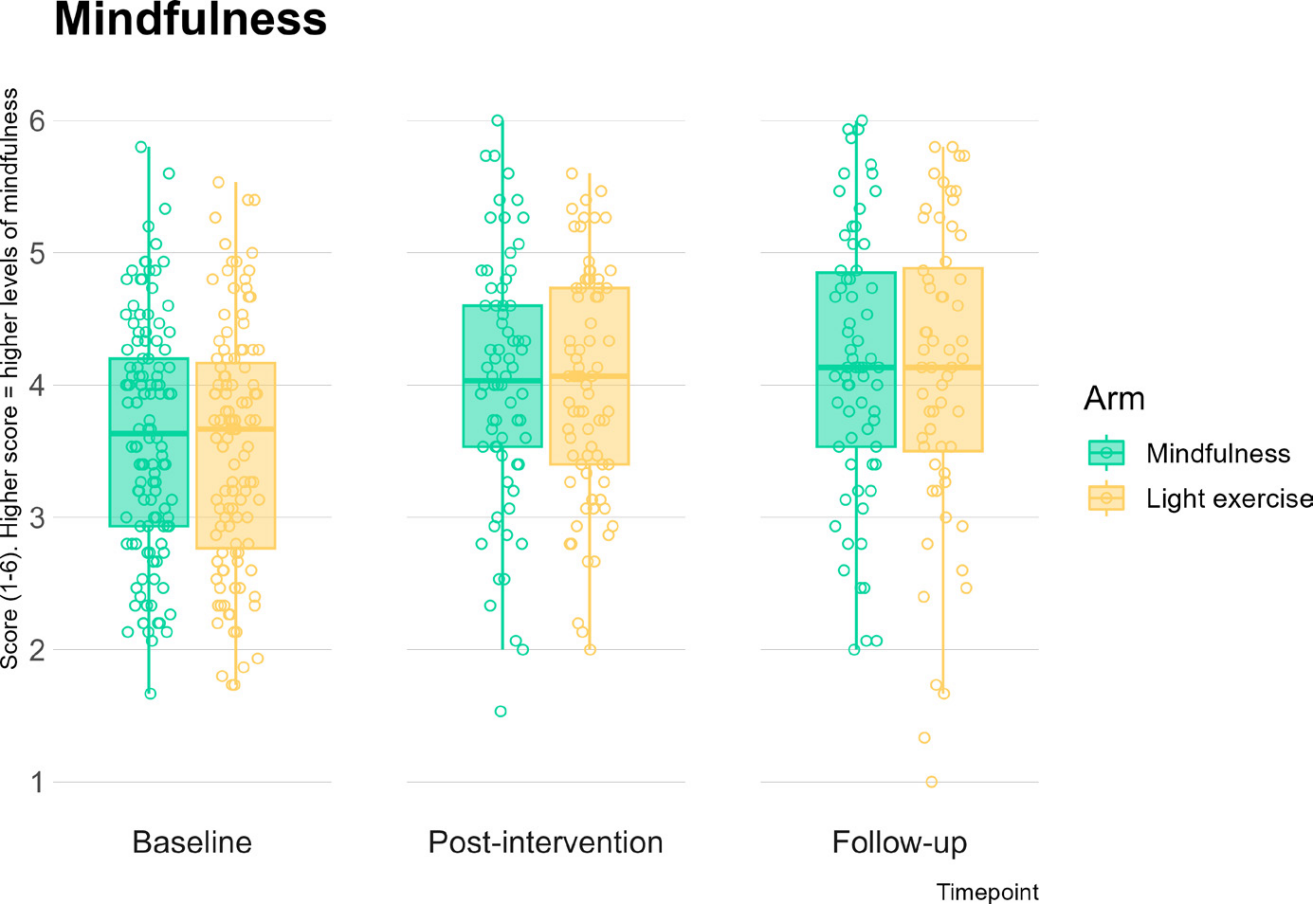
Mindful Attention Awareness Scale (MAAS) score at baseline, postintervention and 12-week follow-up compared across the two study arms.

**Figure 5:**
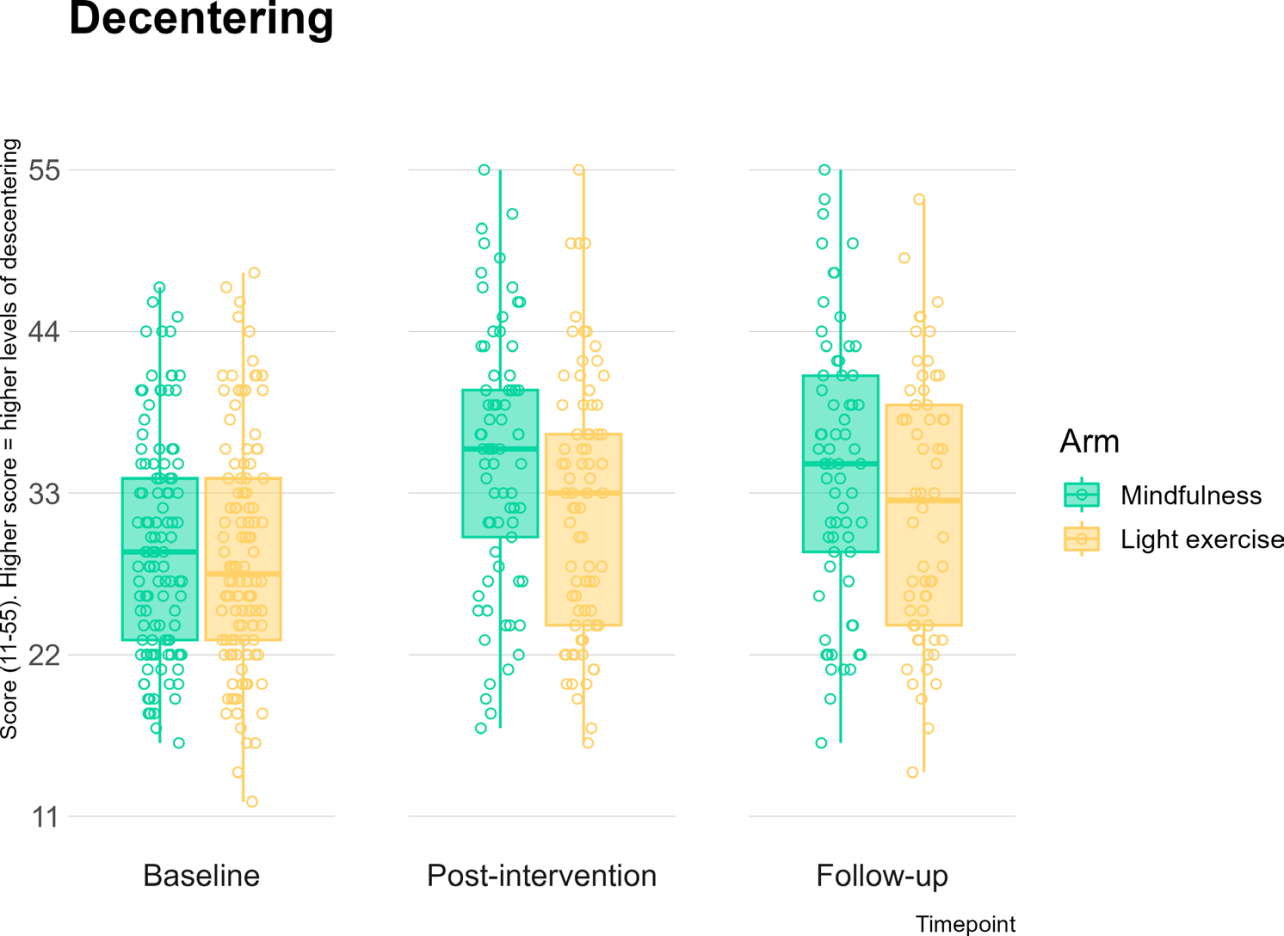
Decentring score at baseline, postintervention and 12-week follow-up compared across the two study arms.

Between-arm improvements at postintervention and follow-up in stress, anxiety, depression and mindful awareness were trivial (*d*s*<*0.10) and not statistically significant, for all time points (figures 3 and 4; online supplemental material 3: table 2). Small effect sizes were seen in favour of the mindfulness arm for decentring at postintervention (*d*=0.24, p=0.07) and at follow-up (*d*=0.22, p=0.09) (figure 5; online supplemental material 3: table 2), although again, these were not significant as expected for a feasibility trial. The remaining effect sizes were smaller than 0.2 and are reported in online supplemental material 3: table 2. All participants demonstrated a significant improvement in mental health outcomes, regardless of intervention allocation: moderate, significant effect sizes were observed for baseline to postintervention change and baseline to follow-up changes across all mental health outcomes (see table 2).

**Table 2:** Baseline to postintervention and baseline to follow-up changes across both arms

Outcome	Preintervention to postintervention						Preintervention to 12-week follow-up					
	Mindfulness		Light exercise		Both arms		Mindfulness		Light exercise		Both arms	
	*d*	P value	*d*	P value	*d*	P value	*d*	P value	*d*	P value	*d*	P value
WRFQ	0.15	0.27	0.12	0.34	0.10	0.28	0.08	0.53	0.15	0.26	0.14	0.12
PSS	−0.86	<0.001	−0.52	<0.001	−0.58	<0.001	−0.40	<0.01	−0.33	<0.05	−0.40	<0.001
GAD-7	−0.52	<0.001	−0.40	0.003	−0.40	<0.001	−0.30	0.02	−0.16	0.21	−0.26	<0.001
PHQ-9	−0.43	<0.01	−0.21	0.11	−0.49	<0.001	−0.54	<0.001	−0.30	0.02	−0.25	<0.001
WSAS	0.65	0.09	0.73	0.04	0.70	<0.05	0.75	0.07	0.62	0.10	0.67	<0.01
Decentring	0.62	<0.001	0.38	<0.01	0.60	<0.001	0.59	<0.001	0.39	<0.01	0.38	<0.001
MAAS	0.48	<0.001	0.36	<0.01	0.46	<0.001	0.45	<0.01	0.39	<0.01	0.37	<0.001

GAD-7, Generalized Anxiety Disorder 7-item Scale; MAAS, Mindful Attention Awareness Scale; PHQ-9, Patient Health Questionnaire 9; PSS, Perceived Stress Scale; WRFQ, Work Role Functioning Questionnaire; WSAS, Work and Social Adjustment Scale.

#### Cognitive control

The assumptions of normality and sphericity were not met for either cognitive task. We therefore analysed the data using linear mixed-effects models.^79 80^ Descriptive statistics and figures are reported in online supplemental table 3: table 4. For the affective stop-signal task, when adjusting for baseline and allowing for random effects for each participant, we found a trivial effect size for the interaction between the intervention and affective condition at both postintervention (beta=−0.82, SE=2.04, t(139.05)=−0.4, p=0.69, *d*=−0.05) and follow-up (beta=−1.16, SE=2.17, t(129.01)=−0.53, p=0.59, *d*=−0.01). Similarly, in the affective learning task, when adjusting for baseline and allowing for random effects for each participant, we found trivial between-arm effect sizes for accuracy at both postintervention (beta=0.001, SE=0.003, t(150.17)=0.34, p=0.74, *d*=0.04) and at follow-up (beta=0.0002, SE=0, t(172.91)=0.09, p=0.93, *d*=0.07).

### Mediation

We used the unimputed data set for mediation analyses. This comprised 43 participants with complete data. We were interested in whether SSRT on negative valence trials at postintervention mediated the effect of group allocation on the WRFQ total score at follow-up. A non-significant, indirect effect was observed (indirect effect: −0.71, p=0.54). The direct effect (0.79, p=0.84) and total effect (0.08, p=0.98) were also non-significant. The statistically non-significant results are to be expected in a feasibility study, while the effect sizes can inform future power analyses in the future trials.

## Conclusions

This randomised controlled feasibility trial demonstrated the acceptability of using an online MBP and a light physical exercise programme in the workplace, as well as the feasibility of conducting a trial to assess the effectiveness of these interventions. Regarding trial feasibility, the attrition rates were similar in both groups, as well as compared with other online trials,^64^ and within the expected attrition rate in our power calculations. However, missingness still constitutes a limitation: it could have counteracted the protection of randomisation against confounding, and compromise the external validity of the findings, which potentially only apply to those willing to complete outcome measures. We found little evidence on cross-arm contamination in the rates of reported mindfulness practice and physical activity. The proportion of eligible employees who chose to partake the study was low, but it reflects workplace well-being uptake in general.^81^

While a full-scale trial is feasible, it is not warranted. The online, self-guided MBP, in comparison to an active control group (light physical exercise) delivered in a similar format, offered negligible additional benefits for work performance either immediately at postintervention or 12 weeks later. Neither mindfulness nor light exercise improved self-rated work performance: we observed minimal effect sizes for preintervention to postintervention within-group change. There was an improvement in day-to-day ratings of work performance across both arms but again no difference between arms. As it was not an efficacy trial, we cannot attribute the size of the effects to the intervention. The MBP used could be efficacious in ideal conditions but may have suffered from low attendance and/or engagement. By capturing the effectiveness in a naturalistic setting, we aimed to mirror the conditions under which such programmes are typically implemented where people are likely to stop attending.^81^ In order to improve the probability that potential intervention effects manifest and are captured, programme designers need to direct efforts to engagement. One way in which they could do this is by involving representatives of the target communities in the design phase of the intervention.^82^ Once the intervention is designed, researchers involved in intervention evaluation could also involve target community representatives in the design of the trial to tailor efforts to retain participants in the assessments.

This feasibility trial is one of the first to compare the effects of MBPs on work performance against an active control group.^35^ Although diverse definitions of work performance have been used across trials, results align between the current trial and the three prior trials using active control groups. Two prior trials comparing an MBP against offering a list of self-help resources found trivial effect sizes for health-related absences^20^ or work engagement.^23^ Similarly, Pipe and colleagues^83^ compared an MBP to a ‘structured educational series’ on stress and leadership strategies and found no statistical differences between arms for caring efficacy in 33 nurses (effect size not reported). Our study therefore contributes to growing evidence that MBPs may offer minimal benefit for improving work performance when compared with an active control group.

Currently, there is no standard measure of work performance which allows for comparison between job roles and industries. While absenteeism and presenteeism could be used for that purpose, owing to their relatively low frequency in generally well populations, a considerable sample size is needed to detect between-arm differences. Use of WRFQ allowed us to recruit participants from various employers and without restricting recruitment to a particular role. While this novel approach would have made the results more applicable across industries and roles, there was some evidence of ceiling effects. Further work to identify appropriate outcome measures may be necessary prior to later phase trials to evaluate work performance, to ensure that study results allow comparison across job roles and industries.

Both the mindfulness and light exercise interventions were anticipated to improve mental health and well-being, and our results demonstrated these benefits. Compared with baseline, there was a statistically significant improvement in stress, anxiety, depression, decentring and mindfulness across both arms at postintervention, with these effects sustained at 12 weeks. The within-group effect sizes in the MBP group were of an expected size compared with effect sizes found in trials with waitlist control groups.^13^ Compared with within-group effect sizes in the MBP arm, the within-group effect sizes in the light physical exercise (LE) group were slightly smaller and in some measures not statistically significant (PHQ-9 at postintervention and GAD-7 at follow-up in the light physical exercise group). Similar to a recent meta-analysis,^11^ we found no evidence for superiority of the MBP over the active control group on mental health outcomes. Indeed, the benefits of exercise for mental health are well established.^51 84^ The light physical exercise programme was effective in controlling for mental health effects of the MBP, which it was designed to do. Our results further suggest that as a low-intensity workplace-based intervention, exercise may yield similar benefits to mindfulness, when delivered in an online, self-guided manner.

Trivial between-arm effect sizes for cognitive control, regardless of affective valence, provide little support for a full-scale study to investigate the beneficial effect of MBPs via cognitive control when controlled for mental health benefits. Much of the prior evidence for a cognitive control pathway is based on trials with passive control groups,^11^ which may indicate that the previously reported effects are driven by non-specific factors. Recent meta-analyses found MBPs not to have cognitive control effects when compared with active control groups.^11 85^ Interestingly, we did observe that decentring might improve more with MBPs than light physical exercise. Decentring has been previously posited as a core mechanism underpinning the effects of mindfulness,^86^ and thus further exploration of decentring may be a more promising avenue for future research.

### Clinical implications

Overall, our results do not support progression to a later phase trial comparing an online MBP to a similarly delivered light exercise course. However, this feasibility trial does provide important insights for future trials of workplace-based interventions. Sample characteristics should be considered. We found higher take-up in the local authorities, which seemingly have lower well-being budgets than the private sector, and may represent an attractive setting for future trials of workplace-based well-being programmes. Although the majority of our participants were self-identifying as women, the majority also reported no caring responsibilities. Future trials and delivery of workplace-based interventions may benefit from exploring how to facilitate engagement for man-identifying employees and carers.

In sum, this feasibility trial indicated that the two interventions used were both acceptable to participants and, based on contamination data, it is feasible to randomise colleagues into different study arms. Yet, online MBPs are unlikely to yield bigger effect sizes than an alternative well-being programme, and indeed may provide little improvement in work performance if at all. Therefore, we found little support for a future superiority trial comparing MBP and light physical exercise. There have been several studies to demonstrate that offering MBPs is better than not doing it (ie, than passive controls)^11 35 85 87^ and our own results indicate that mental health is likely to be improved by an MBP as well as by a light physical exercise course. However, our findings should be considered, along with employee preferences and needs (eg, ability to engage in physical exercise), when purchasing and/or making recommendations about delivery of workplace-based well-being programmes with the specific aim of improving work performance.

10.1136/bmjment-2023-300885.supp2 Supplementary data

10.1136/bmjment-2023-300885.supp3 Supplementary data

## Data Availability

No data are available.
