# Supplementary material for: Work Engagement and Well-being Study (SWELL): a randomised controlled feasibility trial evaluating the effects of mindfulness versus light physical exercise at work
Source: BMJ Ment Health. 2024 Feb 28;27(1):e300885. doi: 10.1136/bmjment-2023-300885 (PMC10910646; doi:10.1136/bmjment-2023-300885)
Supplement: Supplementary data [file bmjment-2023-300885supp004.pdf]

BMJ Publishing Group Limited (BMJ) disclaims all liability and responsibility arising from any reliance placed on this supplemental material which has been supplied by the author(s)

The Work Engagement and Well-being Study (SWELL): A randomised controlled feasibility trial evaluating the effects of mindfulness versus light physical exercise at work

For the purpose of open access, the author has applied a Creative Commons Attribution (CC BY) licence to any Author Accepted Manuscript version arising from this submission.

Supplementary Materials 2: Methods

Outcomes

Primary outcome: Work performance

The WRFQ captures perceived difficulties in meeting work demands. As the WRFQ can be used applied to manual labour to desk-based jobs, it offers variety and comparability across industries and job roles. Items query the ability to focus on work and complete tasks in a timely manner. Items are rated on a 5-point scale ('difficult all of the time' to 'difficult none of the time'), with higher scores indicating better functioning. A 6th option denotes 'does not apply to my job' and was treated as missing when scoring.

We made a number of modifications to the original questionnaire. First, we reframed the questionnaire instructions so as not to instruct people to think of their problems, by omitting the phrase "how much of the work time did your physical health or emotional problems make it difficult for you to do the following?". The modified instructions thus read: "These questions ask you to rate the amount of time during the past four weeks that you had a difficulty handling certain parts of your job. It concerns the hours you worked in the past four weeks. Mark the "does not apply to my job" box only if the question describes something that is not part of your job.". We hoped rephrasing would reduce the risk of ceiling effects. Second, we were concerned that there may be variances in the way people interpret the items and the rating scale. Particularly we found that the framing around difficulties slipped people's mind, so they tended to express a sense of accomplishment and thus tick „most of the time" when things went well. We thus changed the wording on the statements a) to reinforce the fact we are asking about difficulties, b) remind them about time frame and c) changed the wording from the third person

(you) to the first (I). For example, the item „…feel a sense of accomplishment in your work" became „In the past 4 weeks, I had difficulties feeling a sense of accomplishment in my work". We also changed item 2 to reflect the fact many people were still working from home due to the COVID-19 pandemic: "In the past 4 weeks, I had difficulties starting on my job as soon as I arrived at work (or started workday if working from home)".

Modified Work Role Functioning Questionnaire

These questions ask you to rate the amount of time during the past four weeks that you had a difficulty handling certain parts of your job.

It concerns the hours you worked in the past four weeks.

Mark the "does not apply to my job" box only if the question describes something that is not part of your job.

#		Never	Some-times	About half of the time	Most of the time	All of the time	Does not apply for my job	Prefer not to answer
1	In the past 4 weeks, I had difficulties getting going easily at the beginning of the workday	○	○	○	○	○	○	○
2	In the past 4 weeks, I had difficulties starting on my job as soon as I arrived at work (or started workday if working from home)	○	○	○	○	○	○	○
3	In the past 4 weeks, I had difficulties doing my work without stopping to take extra breaks or rests	○	○	○	○	○	○	○
4	In the past 4 weeks, I had difficulties sticking to a routine or schedule	○	○	○	○	○	○	○
5	In the past 4 weeks, I had difficulties working fast enough	○	○	○	○	○	○	○
6	In the past 4 weeks, I had difficulties finishing work on time	○	○	○	○	○	○	○
7	In the past 4 weeks, I had difficulties doing my work without making mistakes	○	○	○	○	○	○	○
8	In the past 4 weeks, I had difficulties satisfying the people	○	○	○	○	○	○	○

Supplemental material BMJ Publishing Group Limited (BMJ) disclaims all liability and responsibility arising from any reliance placed on this supplemental material which has been supplied by the author(s) BMJ Ment Health

	who judge my work							
**9**	In the past 4 weeks, I had difficulties feeling a sense of accomplishment in my work	◯	◯	◯	◯	◯	◯	◯
**10**	In the past 4 weeks, I had difficulties feeling I have done what I am capable of doing	◯	◯	◯	◯	◯	◯	◯
**11**	In the past 4 weeks, I had difficulties lifting, carrying, or moving objects at work weighing more than 10lbs/4.5 kgs	◯	◯	◯	◯	◯	◯	◯
**12**	In the past 4 weeks, I had difficulties sitting, standing, or staying in one position for longer than 15 minutes while working	◯	◯	◯	◯	◯	◯	◯
**13**	In the past 4 weeks, I had difficulties repeating the same motions over and over again while working	◯	◯	◯	◯	◯	◯	◯
**14**	In the past 4 weeks, I had difficulties bending, twisting, or reaching while working	◯	◯	◯	◯	◯	◯	◯
**15**	In the past 4 weeks, I had difficulties using hand-held tools or equipment (for example, a phone, pen, keyboard, computer mouse)	◯	◯	◯	◯	◯	◯	◯
**16**	In the past 4 weeks, I had difficulties keeping my mind on my work	◯	◯	◯	◯	◯	◯	◯
**17**	In the past 4 weeks, I had difficulties doing work carefully	◯	◯	◯	◯	◯	◯	◯
**18**	In the past 4 weeks, I had difficulties concentrating on my work	◯	◯	◯	◯	◯	◯	◯
**19**	In the past 4 weeks, I had difficulties working without losing my train of thought	◯	◯	◯	◯	◯	◯	◯
**20**	In the past 4 weeks, I had difficulties easily reading or using my eyes when working	◯	◯	◯	◯	◯	◯	◯
**21**	In the past 4 weeks, I had difficulties speaking with people in-person, in meetings or on the phone/videocall	◯	◯	◯	◯	◯	◯	◯
**22**	In the past 4 weeks, I had	◯	◯	◯	◯	◯	◯	◯

	difficulties controlling my temper around people when working							
**23**	In the past 4 weeks, I had difficulties setting priorities in my work	◯	◯	◯	◯	◯	◯	◯
**24**	In the past 4 weeks, I had difficulties handling changes in my work	◯	◯	◯	◯	◯	◯	◯
**25**	In the past 4 weeks, I had difficulties processing incoming information, for example e-mails, in time	◯	◯	◯	◯	◯	◯	◯
**26**	In the past 4 weeks, I had difficulties performing multiple tasks and the same time	◯	◯	◯	◯	◯	◯	◯
**27**	In the past 4 weeks, I had difficulties being proactive, show initiative in my work	◯	◯	◯	◯	◯	◯	◯

Secondary outcomes

Selecting IAPS images

To select images to use in the affective Learning Task from the International affective picture system (IAPS (1)), we first chose images that depicted humans as the main subject but excluded images that displayed nudity or were of sexual nature. We then used the affective ratings published as part of the manual (2) to calculate z-score for valence for each image. We divided the z-scores to deciles and assigned deciles 2 and 3 to the negative condition and deciles 5 and 6 to the neutral condition. We discarded images from the remaining deciles.

Affective Stop-Signal Task

At the beginning of each trial within the task, a negative or a neutral image appeared, followed by a go-signal (left or right arrow). Participants needed to respond with a corresponding key press. On a minority of trials (20%), the go-signal was followed by a stop-signal (upwards arrow) in which a go-response was required to be inhibited. When inhibition was successful, the stop-signal delay on the subsequent trial was increased by 20ms. Reaction times (in both, go- and stop-trials), response accuracy (failure or success in inhibiting response) and variability in reaction time throughout the task (a proxy for the ability to overcome errors) were measured.

Affective Probabilistic Reversal Learning Task

The task consisted of 6 phases, 3 forming a neutral condition and 3 forming an emotionally negative condition. Each trial began with a negative or a neural image from the International Affective Picture System (IAPS) (83). Next, pairs of stimuli were presented, and participants were asked to select one item in each pair to gain a reward. In each pair, one of the stimuli was more likely to be rewarded (i.e., reinforced on 80% of trials). Feedback was presented after each response. Through trial-and-error, participants learnt which stimuli are more frequently rewarded. After a certain number of trials (a phase), the contingency of reinforcement switched. In Phase 2, the other stimulus in the pair was more frequently reinforced. In Phase 3, the reinforcement was switched again. Reaction times and response accuracy (i.e., selecting the rewarded member of the pair) were recorded.

Statistical Methods

Missing data were multiply imputed using the *mice* package (80). For questionnaire data, we used predictive mean matching models to impute the total score. Task data were imputed using random forest models as they provided a better fit. We included a large number of variables as predictors (81) during imputation; (a) full scores of the primary outcome, mechanism outcomes,

mental health outcomes, process outcomes and work-related outcomes at all time-points; (b) the rating on the single-item outcomes of work-related outcomes at all time-points and preference of allocation; and (c) programme take-up. The variables for the two arms were imputed separately and then combined for data analysis (82). Imputation was performed for all randomised participants, including those who did not respond to any items at post-intervention or follow-up. We imputed 100 datasets.

Mediation analysis using the *mediation* package (83) tested the hypothesis that mindfulness training, relative to the control intervention, modifies work performance via changes in cognitive control. The outcome was WRFQ total score at follow-up and the mediator was stop-signal reaction time in the negative condition at post-intervention. The predictor variable was the study arm. The statistical analysis plan had pre-specified that only participants who completed at least half of the sessions would be included. However, due to a data collection problem we were not able to verify the number of sessions attended. We therefore included all participants who did at least one session. Daily work performance was evaluated with mixed-effects models, with arm allocation and day as a fixed effects. Participant ID, nested within employer was set as the random effect.

Intervention condition: Be Mindful

The TIDieR (Template for Intervention Description and Replication) Checklist (6) in Supplementary Material 1b serves a guide on where to find information about the Be Mindful programme.

The four-week course consists of 10 sessions, with two sessions completed per week. Sessions include various videos (between 28 s and 7 minutes of length) and text to teach formal meditations as well as informal mindfulness techniques, such as mindful walking and mindful eating. Participants are then asked to practice mindfulness meditation (the type of meditation varies week-to-week and is between 10 and 30 minutes long) and complete an informal exercise (e.g., eating a meal mindfully)

The course draws from Mindfulness-Based Stress Reduction (3) and Mindfulness-Based Cognitive Therapy (4). The course consisted of 2 weekly sessions, first of it longer, the second a shorter top-up. Daily homework included a formal mediation practice with the assistance of video/audio recordings (up to 30 minutes), and one or two informal exercises per day (see Table 1). Twice a week, participants receive generic e-mails motivating them to practice and informing them when the next module is available.

Table 1. Overview of the Be Mindful content

Week/session	Content	Homework
Getting started	Registration; introduction to course; completion of Stress, Anxiety, and Depression assessment	None
**Week 1 – Stepping out of automatic pilot**		
Session 1	Body scan; being mindful doing routine activities; mindful eating	Practice body scan
Session 2	Dealing with barriers	
**Week 2 – Reconnecting with body and breath**		
Session 1	Mindful breathing	Practice mindful breathing; keeping an Event Awareness Journal; practice moving mindfully
Session 2	Physical barometer	
**Week 3 – Working with difficulties**		
Session 1	Breathing space; sitting meditation	Practice breathing space and sitting meditation
Session 2	Thoughts are just thoughts	
**Week 4 – Mindfulness in daily life**		
Session 1	Preparing for stress; reflection on stress strategies	Practice activity awareness, breathing space, and action step; stress strategies
Session 2	Mindful walking	
**Going forward**		
Session 1	Additional resources; completion of Stress, Anxiety, and Depression assessment; completion certificate	None

Control condition: light exercise programme

A detailed behaviour change rationale of the light exercise course is outlined elsewhere (5). The TIDieR (Template for Intervention Description and Replication) Checklist (6) in Supplementary Material 1c serves a guide on where to find information about the Light Exercise Programme.

Participants followed pre-recorded videos that guided them to perform whole-body exercises such as joint rotation and stretching. The programme was not designed to improve strength or cardiovascular fitness and was developed by JG, a public health doctor in collaboration with a body posture expert.

The programme is not manualised. To match the intervention group in the current study, the original programme was extended from five days to seven days to match the frequency of engagement to the intervention condition. There was no new material introduced compared to the original intervention, rather the existing material was combined to create new videos.

The programme consisted of short videos (10 - 12 minutes), one for each day. Each video introduced seven to eight exercises from the list presented in Table 2 providing participants with

a mix of exercises across the whole body. The exercises were presented progressively with easier variants in the first two weeks. Every once in a while, the participants were reminded of the postures needed to conduct the exercises well and were encouraged to modify the exercise when experiencing discomfort.

The video featured four actors (two male, two female, various ages) demonstrating the exercises. Participants accessed the videos online via Qualtrics (7). To match the contact frequency to the Be Mindful intervention programme, the links to the exercises were sent via e-mails twice a week with each e-mail containing links to the daily exercises for the days up to the next e-mail.

Table 2. Exercises in the light exercise programme

**Body part**[a]	**Exercise**
**Neck**	Neck release side-to-side
	Neck release ear-to-shoulder
	Neck release hand-on-ear
**Upper back and shoulders**	Upper back stretch
	Standing shoulder roll
	Shoulder blade contractions
	Shoulder roll
	Front of shoulder and chest stretch
	Rhomboid stretch
	Shoulder shrug
	Interlaced fingers stretch on chair
	Interlaced fingers stretch up and to the side
	Interlaced finger and hamstring stretch
**Back**	Waist twists
	Lower back stretch
	Spinal twist
	Behind your back twist
**Arms**	Elbow curls
	Hand and wrist stretch
	Reaches
	Triceps stretch
	Arm twists
	Arm circles
	Interlaced finger stretch
**Feet, Legs and Hips**	Side lunge
	Squad stretches
	Hamstring stretch

Calf stretch
Hip circles
Quad stretch
Squats
Foot stretch
Foot stretches pedals up on toes
Ankle circles on chair
Ankle circles on ground
Ankle circles on leg

Notes. [a]Some exercises may engage multiple muscle groups, and the categorisation is a generalization based on the primary focus of each exercise.

REFERENCES

1. Bradley MM, Lang PJ. International Affective Picture System. In: Zeigler-Hill V, Shackelford TK, editors. Encyclopedia of Personality and Individual Differences [Internet]. Cham: Springer International Publishing; 2017 [cited 2020 Feb 25]. p. 1–4. Available from: https://doi.org/10.1007/978-3-319-28099-8_42-1
2. Lang PJ, Bradley MM, Cuthbert BN. International affective picture System (IAPS): Affective ratings of pictures and instruction manual [Internet]. Gainesville, FL: University of Florida; 2008 [cited 2023 Jul 11]. Available from: https://www2.unifesp.br/dpsicobio/adap/instructions.pdf
3. Kabat-Zinn J. Full catastrophe living : how to cope with stress, pain and illness using mindfulness meditation / by Jon Kabat-Zinn. Revised an. London : Piatkus, c2013.; 2013.
4. Segal ZV, Williams JMG, Teasdale JD. Mindfulness-based cognitive therapy for depression. 2nd ed. New York: Guilford Press; 2013. 451 p.
5. Galante J, Bekkers MJ, Mitchell C, Gallacher J. Loving-Kindness Meditation Effects on Well-Being and Altruism: A Mixed-Methods Online RCT. Appl Psychol Health Well-Being. 2016 Nov 1;8(3):322–50.
6. Hoffmann TC, Glasziou PP, Boutron I, Milne R, Perera R, Moher D, et al. Better reporting of interventions: template for intervention description and replication (TIDieR) checklist and guide. BMJ. 2014 Mar 7;348:g1687.
7. Qualtrics [Internet]. Provo, Utah, USA: Qualtrics; 2021. Available from: https://www.qualtrics.com
