# Supplementary material for: Work Engagement and Well-being Study (SWELL): a randomised controlled feasibility trial evaluating the effects of mindfulness versus light physical exercise at work
Source: BMJ Ment Health. 2024 Feb 28;27(1):e300885. doi: 10.1136/bmjment-2023-300885 (PMC10910646; doi:10.1136/bmjment-2023-300885)
Supplement: Supplementary data [file bmjment-2023-300885supp005.pdf]

The Work Engagement and Well-being Study (SWELL): A randomised controlled feasibility trial evaluating the effects of mindfulness versus light physical exercise at work

For the purpose of open access, the author has applied a Creative Commons Attribution (CC BY) licence to any Author Accepted Manuscript version arising from this submission.

SUPPLEMENTARY MATERIALS 3: FINDINGS

Preferences and contamination

Supplementary Table 1. Preferences and contamination

				Mindfulness		Light exercise	
**Outcome**		**Timepoint**	**Item**	**n**	**%/m (sd)**	**n**	**%/m (sd)**
Engaged in other arm's practice		Post-intervention	Not at all	15	(12.3%)	34	(28.57%)
			A few times	33	(27.05%)	35	(29.41%)
			Often	27	(22.13%)	8	(6.72%)
			Missing	47	(38.52%)	42	(35.29%)
Experiences with meditation	Practiced mindfulness formally	Post-intervention	Not at all	7	(5.74%)	22	(18.49%)
			Less than half an hour a week	17	(13.93%)	11	(9.24%)
			Between 0.5 and 1 hour a week	23	(18.85%)	4	(3.36%)

				Mindfulness		Light exercise	
**Outcome**		**Timepoint**	**Item**	**n**	**%/m (sd)**	**n**	**%/m (sd)**
			Between 1 and 3 hours a week	27	(22.13%)	5	(4.2%)
			More than 3 hours a week	1	(0.82%)	1	(0.84%)
			Missing	47	(38.52%)	76	(63.87%)
		Follow-up	Not at all	43	(35.25%)	38	(31.93%)
			Less than half an hour a week	10	(8.2%)	7	(5.88%)
			Between 0.5 and 1 hour a week	9	(7.38%)	10	(8.4%)
			Between 1 and 3 hours a week	4	(3.28%)	5	(4.2%)
			More than 3 hours a week	1	(0.82%)	1	(0.84%)
			Missing	55	(45.08%)	58	(48.74%)
	Practiced mindfulness informally	Post-intervention	Never	2	(1.64%)	6	(5.04%)
			Rarely	3	(2.46%)	7	(5.88%)
			Sometimes	41	(33.61%)	19	(15.97%)
			Often	19	(15.57%)	9	(7.56%)
			Very often	10	(8.2%)	2	(1.68%)
			Missing	47	(38.52%)	76	(63.87%)
		Follow-up	Never	10	(8.2%)	17	(14.29%)
			Rarely	20	(16.39%)	8	(6.72%)
			Sometimes	18	(14.75%)	28	(23.53%)
			Often	13	(10.66%)	5	(4.2%)
			Very often	6	(4.92%)	3	(2.52%)
			Missing	55	(45.08%)	58	(48.74%)
Preference of intervention[a]		Post-intervention		75	1.24 (36.37)	77	-12.04 (28.32)
Talking about own training with colleagues in other arm[b]		Post-intervention		75	5.07 (14.63)	77	2.81 (5.85)

			Mindfulness		Light exercise	
**Outcome**	**Timepoint**	**Item**	**n**	**%/m (sd)**	**n**	**%/m (sd)**
Weekly moderate intensity exercise (min)	Baseline		122	359.9 (310.21)	119	350.12 (298.25)
	Post-intervention		73	406.19 (288.98)	76	399.71 (322.19)
	Follow-up		61	380.85 (282.78)	61	379.77 (255.89)

[a]Scale: -50 (stong preference towards MBP) to 50 (strong preference towards LE); [b]Scale: 0 (not even once) to 100 (almost daily);

BMJ Publishing Group Limited (BMJ) disclaims all liability and responsibility arising from any reliance placed on this supplemental material which has been supplied by the author(s)

Questionnaire-based outcomes

Supplementary Table 2. Observed questionnaire-based outcomes at all time points

		Mindfulness		Light Exercise		Total			
**Outcome[a]**	**Timepoint**	**n**	**m (sd)**	**n**	**m (sd)**	**n**	**m (sd)**	***d***	***p***
WRFQ	Baseline	66	77.4 (11.85)	53	77.32 (13.84)	119	77.37 (12.72)		
	Post-intervention	36	82.43 (11.6)	32	80.18 (13.56)	-	-	0.06	0.63
	Follow-up	33	82.49 (13.25)	29	78.26 (18.21)	-	-	0.02	0.91
PSS	Baseline	122	20.5 (6.18)	118	20.69 (6.74)	240	20.59 (6.45)		
	Post-intervention	74	16.76 (7.07)	78	17.22 (6.91)	-	-	0.04	0.73
	Follow-up	67	16.9 (7.53)	61	16.52 (6.88)	-	-	-0.01	0.92
GAD-7	Baseline	122	6.48 (4.3)	119	6.27 (4.78)	241	6.37 (4.53)		
	Post-intervention	75	5.23 (4.35)	78	5.05 (4.11)	-	-	-0.00	0.99
	Follow-up	66	5.23 (4.9)	61	4.84 (4.29)	-	-	-0.07	0.58
PHQ-9	Baseline	117	8.21 (4.6)	119	9.12 (5.06)	236	8.67 (4.85)		
	Post-intervention	75	6.37 (4.76)	78	6.58 (5.02)	-	-	0.03	0.82
	Follow-up	66	6.86 (5.85)	61	6.13 (4.72)	-	-	-0.09	0.47
WSAS	Baseline	119	1.76 (4.75)	117	1.95 (5.52)	236	1.85 (5.14)		
	Post-intervention	71	1.54 (4.9)	77	3.35 (7.41)	-	-	0.17	0.18
	Follow-up	65	3.68 (8.41)	59	2.20 (5.11)	-	-	0.00	0.99
Decentering	Baseline	118	29.25 (7.41)	116	28.84 (7.84)	234	29.05 (7.61)		
	Post-intervention	73	35.33 (8.74)	77	32.04 (8.77)	-	-	0.24	0.07
	Follow-up	66	34.7 (9.18)	60	32.05 (9.05)	-	-	0.22	0.09

			Mindfulness		Light Exercise		Total			
**Outcome[a]**		**Timepoint**	**n**	**m (sd)**	**n**	**m (sd)**	**n**	**m (sd)**	***d***	***p***
MAAS		Baseline	122	3.61 (0.88)	115	3.53 (0.91)	237	3.57 (0.9)		
		Post-intervention	72	4.03 (0.93)	77	3.98 (0.86)	-	-	0.02	0.85
		Follow-up	66	4.16 (1.01)	60	4.08 (1.15)	-	-	0.02	0.87
Job importance		Baseline	121	2.43 (0.75)	117	2.28 (0.81)	238	2.36 (0.78)		
		Post-intervention	74	2.3 (0.77)	77	2.42 (0.66)	-	-	-0.14	0.27
		Follow-up	67	2.24 (0.65)	61	2.31 (0.85)	-	-	-0.07	0.56
Overtime (hrs)		Baseline	122	3.82 (6.99)	119	3.29 (5.5)	241	3.56 (6.29)		
		Post-intervention	75	3.58 (5.96)	77	4.32 (5.44)	-	-	0.22	0.09
		Follow-up	66	3.18 (6.08)	61	2.98 (4.51)	-	-	-0.01	0.95
Experiencing health problems, n (%)	No problems	Baseline	98	(80.33%)	100	(84.03%)	198	(82.16%)		
	No problems	Post-intervention	61	(50%)	58	(48.74%)	-	-		
	No problems	Follow-up	49	(40.16%)	48	(40.34%)	-	-		
Experiencing health problems, n (%)	Some problems	Baseline	22	(18.03%)	17	(14.29%)	39	(16.18%)		
		Post-intervention	11	(9.02%)	19	(15.97%)	-	-	0.20[b]	0.11
		Follow-up	16	(13.11%)	12	(10.08%)	-	-	-0.11[b]	0.41
	Missing	Baseline	2	(1.64%)	2	(1.68%)	4	(1.66%)		
		Post-intervention	50	(40.98%)	42	(35.29%)	-	-		
		Follow-up	57	(46.72%)	59	(49.58%)	-	-		

Notes. [a]Abbreviations: WRFQ = work role functioning scale, PSS = perceived stress scale, GAD-7 = generalised anxiety scale, PHQ-9 = Patient Health Questionnaire-9, WSAS = work and social adjustment scale, MAAS = mindful attention awareness scale; [b]Compared to reporting no problems, adjusted for baseline;

Cognitive control

Supplementary Table 3. Observed cognitive control outcomes at all time points

				Mindfulness		Light exercise		Total	
**Outcome**	**Valence**		**Timepoint**	**n**	**m (sd)**	**n**	**m (sd)**	**n**	**m (sd)**
aSST	Negative	Missed go trials (%)	Baseline	101	20.96 (19.37)	101	18.59 (16.61)	202	19.77 (18.04)
			Post-intervention	58	19.05 (20.58)	62	18.04 (19.27)	-	-
			Follow-up	53	16.84 (17.93)	48	15.88 (20.42)	-	-
		Accuracy in go trials (%)	Baseline	101	83.73 (4.54)	101	84.67 (3.56)	202	84.2 (4.1)
			Post-intervention	58	83.33 (4.02)	62	83.97 (4)	-	-
			Follow-up	53	82.41 (11.9)	48	84.4 (5.09)	-	-
		Reaction time in go trials (ms)	Baseline	101	729.17 (98.02)	101	735.63 (96.7)	202	732.4 (97.17)
			Post-intervention	58	738.53 (105.11)	62	736.83 (99.42)	-	-
			Follow-up	53	739.76 (104.34)	48	735.12 (102.39)	-	-
		Probability of responding in stop-signal trials (%)	Baseline	101	58.52 (9.28)	101	59.22 (8.21)	202	58.87 (8.75)
			Post-intervention	58	57.01 (8.51)	62	57.15 (8.18)	-	-
			Follow-up	53	57.09 (10.12)	48	57.71 (7.81)	-	-
		Stop Signal Delay (ms)	Baseline	101	479.8 (134.92)	101	483.98 (116.15)	202	481.89 (125.59)
			Post-intervention	58	497.7 (139.8)	62	505.44 (122.47)	-	-
			Follow-up	53	492.01 (132.69)	48	499.08 (122.89)	-	-
		Reaction time in failed stop-trials (ms)	Baseline	101	232.62 (75.13)	101	213.21 (63.67)	202	222.91 (70.14)
			Post-intervention	58	219.85 (69.61)	62	214.14 (62.7)	-	-
			Follow-up	53	214.08 (50.4)	48	197.74 (59.38)	-	-
		Stop Signal Reaction Time (ms)	Baseline	101	267.59 (63.02)	101	262.73 (65.08)	202	265.16 (63.94)
			Post-intervention	58	260.4 (60.48)	62	252.17 (43.49)	-	-
			Follow-up	53	255.64 (66.5)	48	248.23 (43.81)	-	-

				Mindfulness		Light exercise		Total	
**Outcome**	**Valence**		**Timepoint**	**n**	**m (sd)**	**n**	**m (sd)**	**n**	**m (sd)**
	Neutral	Missed go trials (%)	Baseline	102	20.1 (18.42)	105	19.68 (17.67)	207	19.89 (18)
			Post-intervention	55	17.47 (20.18)	62	18.46 (20.35)	-	-
			Follow-up	54	18.69 (18.48)	50	18.71 (21.92)	-	-
		Accuracy in go trials (%)	Baseline	102	84.05 (3.86)	105	84.32 (3.56)	207	84.19 (3.7)
			Post-intervention	55	84.38 (4.35)	62	84.32 (5.18)	-	-
			Follow-up	54	82.14 (12.5)	50	84.17 (4.79)	-	-
		Reaction time in go trials (ms)	Baseline	102	736.53 (91.39)	105	743.56 (96.51)	207	740.1 (93.86)
			Post-intervention	55	734.28 (100.78)	62	738.55 (97.41)	-	-
			Follow-up	54	738.83 (104.04)	50	744.35 (105.71)	-	-
		Probability of responding in stop-signal trials (%)	Baseline	102	58.22 (8.63)	105	58.6 (7.23)	207	58.42 (7.93)
			Post-intervention	55	59.64 (7.72)	62	59.09 (7.17)	-	-
			Follow-up	54	58.13 (8.2)	50	59 (7.54)	-	-
		Stop Signal Delay (ms)	Baseline	102	478.66 (129.44)	105	492.73 (113.96)	207	485.8 (121.74)
			Post-intervention	55	486.89 (135.23)	62	501.75 (120.89)	-	-
			Follow-up	54	491.86 (130.17)	50	512.17 (130.79)	-	-
		Reaction time in failed stop-trials (ms)	Baseline	102	221.08 (69.16)	105	216.97 (65.92)	207	218.99 (67.4)
			Post-intervention	55	216.42 (74)	62	212.7 (58.89)	-	-
			Follow-up	54	215.01 (58.72)	50	207.21 (68.04)	-	-
		Stop Signal Reaction Time (ms)	Baseline	102	277.96 (57.28)	105	267.41 (64.79)	207	272.61 (61.28)
			Post-intervention	55	261.2 (68.8)	62	248.14 (52.16)	-	-
			Follow-up	54	260.32 (75.46)	50	246.51 (53.14)	-	-
aLT	Negative	Reaction Time (ms)	Baseline	118	697.51 (161.77)	115	691.23 (154.96)	233	694.41 (158.13)
			Post-intervention	65	687.58 (177.18)	68	641.92 (153.52)	-	-
			Follow-up	61	650.8 (179.57)	58	633.07 (145.98)	-	-

				Mindfulness		Light exercise		Total	
Outcome	Valence		Timepoint	n	m (sd)	n	m (sd)	n	m (sd)
		Accuracy (%)	Baseline	118	55.13 (7.39)	115	53.81 (7.52)	233	54.48 (7.47)
			Post-intervention	65	56.23 (7.39)	68	57.38 (7.96)	-	-
			Follow-up	61	57.69 (8.44)	58	57.98 (9.03)	-	-
	Neutral	Reaction Time (ms)	Baseline	118	694.47 (159.65)	115	680.54 (151.25)	233	687.6 (155.38)
			Post-intervention	65	679.28 (172.09)	68	649.72 (144.21)	-	-
			Follow-up	61	655.5 (178.72)	58	631.5 (147.1)	-	-
		Accuracy (%)	Baseline	118	54.86 (7.72)	115	54.84 (8.6)	233	54.85 (8.15)
			Post-intervention	65	55.5 (7.63)	68	56.33 (8.9)	-	-
			Follow-up	61	58.45 (8.98)	58	57.57 (9.27)	-	-

Notes. [a]aSST = affective stop-signal task, aLT = affective learning task

SWELL Study | Supplementary Materials 3

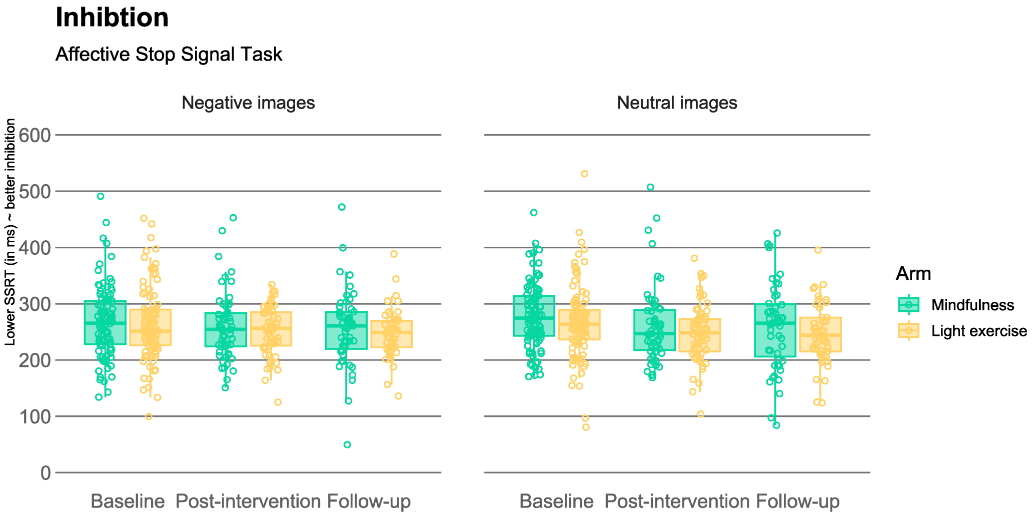

Supplementary Figure 1. Affective Stop Signal Task results at baseline, post-intervention, and follow-up

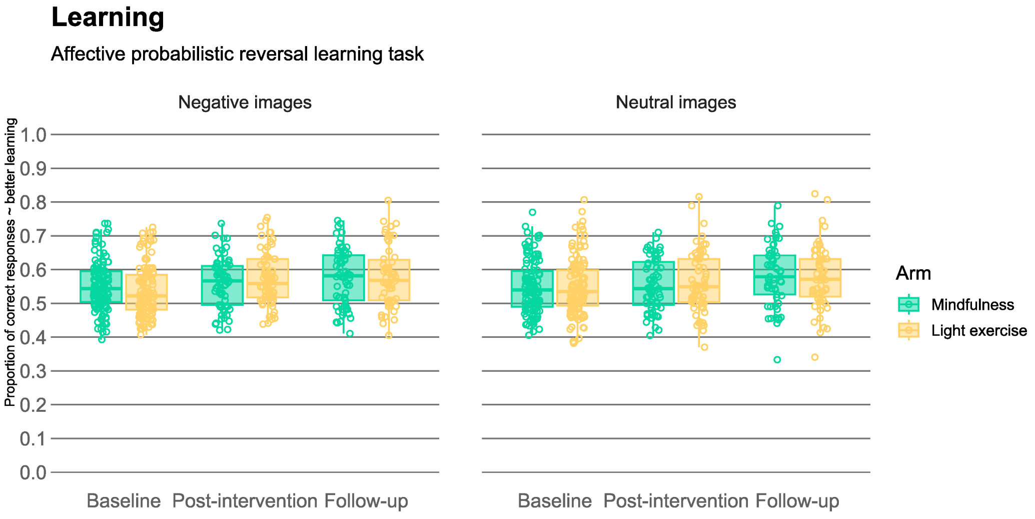

Supplementary Figure 2. Affective Probabilistic Learning Task results at baseline, post-intervention, and follow-up

SWELL Study | Supplementary Materials 3

Daily monitoring

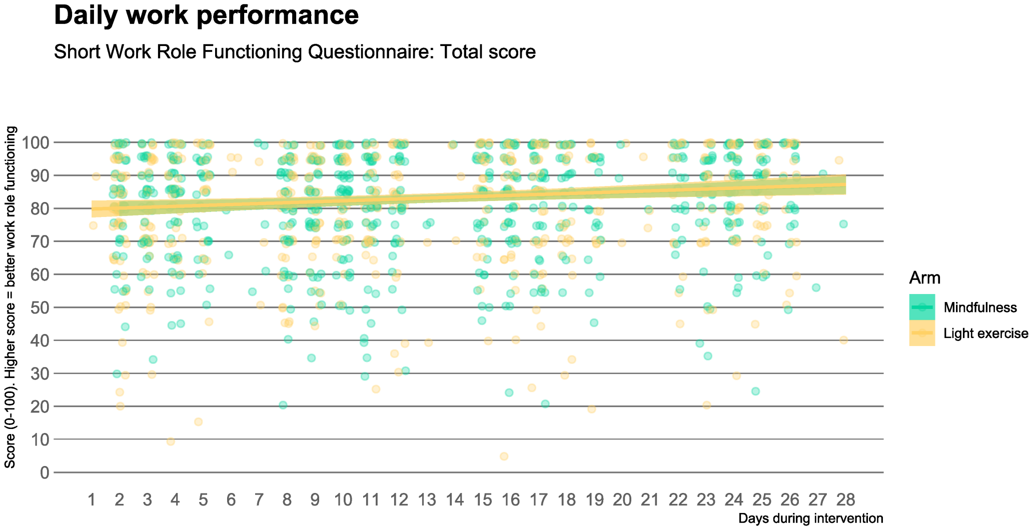

Supplementary Figure 3. Daily monitoring of work role functioning
